# Unilateral vocal nerve resection alters neurogenesis in the avian song system in a region-specific manner

**DOI:** 10.1371/journal.pone.0256709

**Published:** 2021-08-31

**Authors:** Jake V. Aronowitz, Alice Perez, Christopher O’Brien, Siaresh Aziz, Erica Rodriguez, Kobi Wasner, Sissi Ribeiro, Dovounnae Green, Farhana Faruk, Carolyn L. Pytte

**Affiliations:** 1 Psychology Department, Queens College, City University of New York, Flushing, NY, United States of America; 2 Psychology Department, The Graduate Center, City University of New York, New York, NY, United States of America; 3 Biology Department, The Graduate Center, City University of New York, New York, NY, United States of America; Texas Christian University, UNITED STATES

## Abstract

New neurons born in the adult brain undergo a critical period soon after migration to their site of incorporation. During this time, the behavior of the animal may influence the survival or culling of these cells. In the songbird song system, earlier work suggested that adult-born neurons may be retained in the song motor pathway nucleus HVC with respect to motor progression toward a target song during juvenile song learning, seasonal song restructuring, and experimentally manipulated song variability. However, it is not known whether the quality of song per se, without progressive improvement, may also influence new neuron survival. To test this idea, we experimentally altered song acoustic structure by unilateral denervation of the syrinx, causing a poor quality song. We found no effect of aberrant song on numbers of new neurons in HVC, suggesting that song quality does not influence new neuron culling in this region. However, aberrant song resulted in the loss of left-side dominance in new neurons in the auditory region caudomedial nidopallium (NCM), and a bilateral decrease in new neurons in the basal ganglia nucleus Area X. Thus new neuron culling may be influenced by behavioral feedback in accordance with the function of new neurons within that region. We propose that studying the effects of singing behaviors on new neurons across multiple brain regions that differentially subserve singing may give rise to general rules underlying the regulation of new neuron survival across taxa and brain regions more broadly.

## Introduction

New cells that are produced postnatally generally have predetermined lifespans, varying by tissue type, and largely independent of the experience of the cell. In the healthy adult human, epithelial cells survive less than 2 weeks [1] white blood cells live for about 13–20 days, red blood cells survive 100–120 days [2], and liver hepatocytes turnover about every 200–300 days [3,4]. On the other hand, across species and brain regions, lifespans of neurons born in the postnatal brain are highly variable. More interestingly, lifespans differ not only by neuron type, but are dramatically influenced by the cell’s activity and experience, setting them apart in this way from other continually regenerating cell types [5–14].

**Fig 1:**
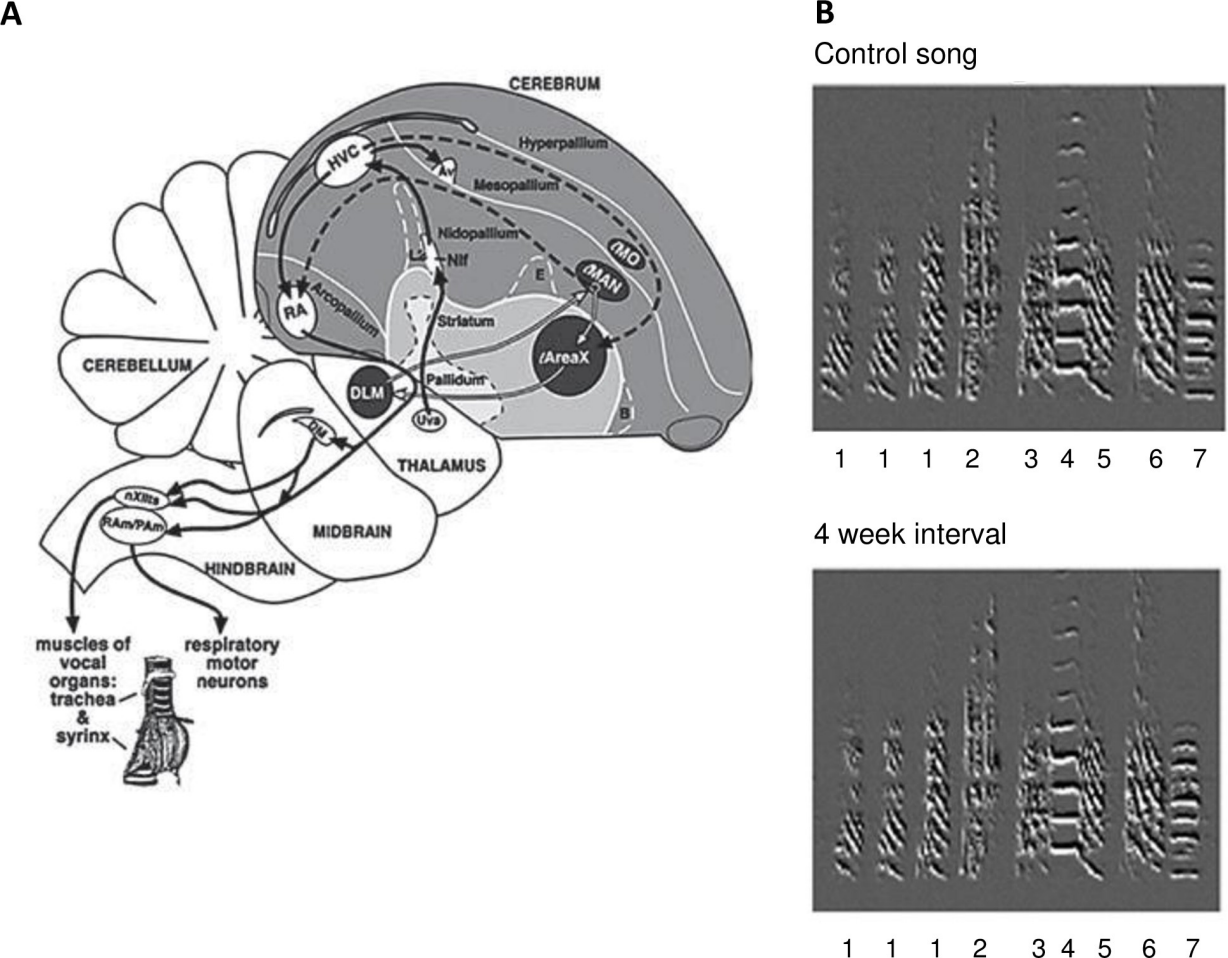
Song system and song stability. **A.** Diagram of the song system in a sagittal section, showing the vocal motor pathway (black solid lines) and the anterior forebrain pathway (dashed and white lines). White lines show the feedback loop between the striatum, thalamus, nidopallium, and projection back to the striatum. AV = Nucleus avalanche; B = nucleus basorostralis; DM = dorsal medial nucleus; DLM = dorsal lateral nucleus of the medial thalamus; E = entopallium; LMAN = lateral magnocellular nucleus of the anterior nidopallium; LMO = lateral oval nucleus of the mesopallium; Nif = interfacial nucleus of the nidopallium; PAm = para-ambiguus; Ram = nucleus retroambiguus; Uva, nucleus uvaeformis; nXIIts = tracheosyringeal portion of the hypoglossal nucleus. Reproduced from Pytte et al. (2011) [42]; syrinx modified from Goller and Suthers (1996) [62]. **B.** Spectral derivatives of a song motif generated with Sound Analysis Pro 2011 [56] showing example song stability over a 4 week interval in a young adult bird (5 mos old). Numbers identify different song elements based on qualitative assessment of acoustic structure, shown here to highlight similarity of structure in the two recordings; number “1” is a repeated introductory syllable. Modified from Pytte et al., (2012) [65].

In songbirds, new neurons are incorporated into regions of the song system that function in song learning, memory, perception, and song production (Fig 1A). These regions make well-defined and different contributions to song behaviors, therefore provide a system for comparing effects of behavioral feedback on new neuron survival across functionally distinct areas within the same individuals.

Behavioral factors affecting new neuron survival have been studied most systematically in HVC [15,16], a sensorimotor nucleus in which about half of the population of new neurons project to the premotor robust nucleus of the arcopallium (RA), becoming part of the motor pathway for song production (HVC_RA_) [17–19]. In the canary, song production positively correlates with new neuron survival [20–22]. This association is mediated by BDNF and testosterone, both of which directly increase new neuron survival [16,23–27]. Housing conditions also impact the lifespan of both HVC_RA_ neurons and new neurons in the auditory region caudomedial nidopallium (NCM), with more new neurons seen in zebra finches or canaries housed in groups compared to singly housed birds [28–31]. Notably, Shevchouk et al., (2017) [29] showed that new neuron proliferation and the subsequent survival of new neurons in HVC can be independently affected by male- or female-paired housing. Numerous behaviors may underlie this effect, including increased singing and increased exposure to songs. NCM affects HVC activity indirectly [32] and perhaps is a source of auditory input into the song system. In addition to HVC and NCM, the basal ganglia nucleus Area X also continues to receive new neurons throughout adulthood and processes song-related sensory feedback [33]. Area X receives projections from a population of non-neurogenic HVC neurons (HVC_X_) and is part of the anterior forebrain pathway necessary for juvenile song learning [34,35] and adult song plasticity [36–38]. Behavioral factors that may influence new neuron numbers in Area X have not yet been investigated. Given the functional and anatomical connections between HVC, NCM and Area X, we explored effects of behavioral feedback on new neurons across these regions.

During embryonic brain development, neuronal activity may contribute to cell survival, whereas lack of activity increases culling, putatively improving network efficiency. Perhaps a similar process continues in adulthood in regions that receive new neurons, such that pathway activity influences the survival of newly incorporated neurons, improving circuit function [39]. This idea is the basis for a model in which new HVC neurons audition for a spot in the song production pathway and get the part when their contribution improves song performance [15,40]. This model considers not only the amount of songs produced, but also the ongoing quality of the song. In this way, accurate progression in a goal-directed behavior (accurate song production) interacts with number of iterations of songs produced in promoting new neuron retention [15,41].

Consistent with this idea, experimentally altered song structure by reversible paralysis of the syringeal muscles resulted in a positive correlation between numbers of new neurons in HVC and song recovery [42]. However, in this work song recovery was confounded with song quality. Higher quality songs improved more, and more quickly, thus had higher values of recovery [42]. Therefore, either song quality *per se* (regardless of song improvement), or song recovery (requiring improvement) may have influenced new neuron survival.

To pull apart these confounded variables, here we tested whether song quality without recovery impacts new neuron survival. To do this, we produced a stable, irreversible, aberrant song by unilaterally denervating the syrinx in adult male zebra finches by sectioning either the left or right tracheosyringeal nerve (nXIIts, called here “TS”). This allowed us to determine directly whether song quality impacts neuronal culling or survival, and also to broaden our investigation to song regions outside of HVC.

We also speculated that distinct effects of feedback on new neurons may occur not only across brain regions, but also perhaps between hemispheres in a given brain region. More new neurons are added to left NCM than right NCM of adult zebra finches [43]. Moreover, the degree of this asymmetry was positively correlated with the quality of song learning and the strength of neuronal memory for songs [43]. Inter-hemisphere comparisons between other brain regions have not yet been conducted. Improving our understanding of how new neuron survival is regulated across brain regions and between hemispheres may inform strategies that consider interconnected effects of a whole brain system in enhancing the survival of new neurons post brain injury or disease as well as in the healthy brain [44–46].

## Materials and methods

### Animals

All methods were approved by the Queens College Institutional Animal Care and Use Committee (protocol #165). Only male zebra finches sing; therefore, we used adult males (14 controls, 17 left TS-cut, 18 right TS-cut). The study was conducted with multiple cohorts, differing in targeted brain regions. Therefore, sample sizes differ depending on the brain region and new neuron marker (DCX+ or BrdU+) and are reported in the figure captions.

Birds were hatched in either the Queens College or Wesleyan University breeding colony and kept with their parents until 90 days of age. Thereafter, birds were group housed, with experimental and control birds housed together in group cages, with auditory and visual interaction with colony birds of both sexes throughout the study. Siblings from the same clutch (within a few days of age) were distributed across the 3 treatment groups when there were 3 or 6 males within a clutch. When equal distribution into treatment groups was not possible within a clutch, individuals were matched across treatment groups across same-aged clutches. Birds were between 4–11 months of age at the time of BrdU injections. We also matched nest of origin across groups, using sequential clutches matched across experimental cohorts. Birds were maintained on a 12:12 h light:dark schedule with food and water available *ad libitum*.

### Song recording and analysis

Song changes after unilateral syringeal denervation have been well documented [47–49]. It is also known that the fine structure of adult song is highly, although not exactly, stereotyped over months, and more so over a 1 week interval as was used in the present study [Fig 1B, 50–53]. TS-cuts can trigger central changes over longer durations of denervation (> 1 week) [47,48,54,55] and we sought to avoid this effect by using a 1 week survival post TS-cut. Because the focus of our study was on the effects of denervation on new neurons and not on documenting the changes in song structure, we only conducted minimal recordings (controls = 5, left TS-cut = 5, right TS-cut = 4).

Adult male zebra finches were temporarily housed individually in sound attenuated chambers in order to record songs pre- and post-surgery. They were recorded 2–4 days prior to surgery, and again within post-operative days 2–4. After recording, the birds were returned to their home cages. Recordings were made using cardioid microphones (Earthworks SR20) and sound-activated Avisoft Recorder (Avisoft Bioacoustics). Zebra finches have a repertoire of a single song type that consists of a variable number of repetitions of an introductory note followed by repeating sequences of generally about 4 to 10 acoustic elements in a consistent order called a “motif.” Recordings of songs were edited to a single motif using Raven sound analysis software (Cornell Lab of Ornithology).

We used two measures to document post-operative changes to the motif: (1) accuracy and (2) percentage of similarity (termed here “similarity”) using Sound Analysis Pro 2011 [56]. The accuracy score indicates the fidelity of song elements by comparing 1 ms song segments between pre- and post-operative motifs. Similarity indicates sameness of song structure at longer intervals (~50 ms) (http://soundanalysispro.com/manual-1). We compared 10 pre- and post-operative motif pairs for each bird and used the means of each of these scores as a measure of postoperative change in song acoustic structure. Pre- and post-operative motif pairs were selected to be identical in song element number and sequence. We used a 10 x 10 comparison matrix such that every motif exemplar was compared with each of the 9 others and redundant comparisons removed from the analysis. We then compared pre-to-post motif accuracy and similarity scores with numbers of new neurons. We also conducted linear regressions between the following measures of pre- to post-operative song change and new neurons counts: accuracy, similarity, difference in pitch, difference in frequency modulation (FM), difference in entropy, difference in goodness of pitch (a measure of periodicity) and difference in amplitude modulation (AM) (see definitions in http://soundanalysispro.com/manual-1). Singing rates were not calculated because the length of recordings were not sufficient in the already small sample size of birds that were recorded to accurately gauge individual singing rates or effects of treatment on song output.

### Bromodeoxyuridine (BrdU) injections and tracheosyringeal denervation

All birds received intramuscular injections of 5-Bromo-2’-deoxyuridine (BrdU; 74 μg/g, pH 7.4, Sigma) 3x/day for three days to label mitotically active cells. Unilateral tracheosyringeal denervation surgeries were performed over 2 days, 21–22 days after the last BrdU injection, such that new neurons were 21–24 days old when song feedback was altered. This time frame was selected in order to ensure new neurons had migrated into the three regions of interest prior to nerve resection.

Birds were anesthetized with either a mixture of ketamine and xylazine (0.03–0.05 mg/g and 0.06 mg/g, respectively), or isoflurane in air (3%, Henry Schein). There were no differences in mean densities of new neurons in HVC, Area X, or NCM (hemispheres combined) between animals anesthetized with either mechanism (2 factor ANOVA, repeated measure on brain region, controls only: F = 0.12, p = 0.733, all treatments combined: F = 0.08, p = 0.778).

Using a surgical microscope (Zeiss Universal S3B), a 3–4 mm rostro-caudal incision was made < 1 mm lateral to the ventral midline to expose the tracheosyringeal nerve. A 2–4 mm section of either the left or right tracheosyringeal nerve was resected to prevent regrowth following surgery. Sham birds received all surgical manipulations except nerve cuts.

### Histology

Birds experienced altered song for seven days and then were overdosed with sodium pentobarbital (Euthasol) and transcardially perfused with 0.1 M phosphate buffered saline (PBS, pH 7.4) followed by 4% paraformaldehyde (PFA; Sigma-Aldrich; pH 7.4). TS nerve sections were confirmed following the perfusion. Brains were post-fixed for 1 h in 4% PFA, rinsed in PBS for 3 h and embedded in polyethylene glycol (MW = 1500; Polysciences). Six-ųm sagittal sections were cut on a rotary microtome. The brain was oriented with the midline parallel to the blade edge. The first complete section through the telencephalon was saved and subsequently every sixth section of tissue was mounted onto Superfrost Plus + slides (VWR). All series were stored at -20°C prior to processing using immunohistochemistry (IHC).

### DCX immunohistochemistry

Sections were brought to room temperature in tris buffered saline (TBS), followed by a 10-min wash in fresh TBS. Sections were then incubated for 30-min in a H₂O₂ solution (97% TBS, 1% methanol, 2% of 3% retail H₂O₂) to eliminate endogenous peroxidases. After three 5-min TBS rinses, non-specific binding was blocked with 5% normal horse serum (Jackson ImmunoResearch Laboratories Inc.) and 0.5% Triton X-100 (Sigma-Aldrich) in TBS for 30-min at room temperature. This was followed by exposure to anti-DCX antibody (goat polyclonal IgG, Santa Cruz Biotechnology; sc-8066, 1:150; or rabbit polyclonal IgG, Abcam; ab18723, 1:1000) in the same blocking buffer overnight at 4˚C. After three 5-min TBS rinses, sections were incubated for 3 h in biotinylated horse anti-goat (1:200, Vector Labs) or biotinylated goat anti-rabbit secondary antibody (1:200, Vector Labs) in TBS, rinsed again, and exposed for 1 h to an avidin-biotin complex (Vector Labs). Sections were rinsed in TBS and then reacted in a solution of 0.04% of 3,3’-diaminobenzidine tetrahydrochloride (DAB, Vector Labs) with nickel until the tissue changed color (~3–10 min). Following three 5-min TBS rinses, sections were dehydrated in ethanols, delipidized in xylenes, and cover slipped with Krystalon (Millipore-Sigma).

### BrdU/Hu immunohistochemistry

Sections were brought to room temperature in 0.1 M phosphate buffer (PB; pH 7.4), then exposed to citrate buffer (pH 5.6–6.0) at 90–95°C for 10 min, followed by a 5-min PB wash (37°C), 3 min in a solution of 0.28% pepsin in 400 ml 0.1 M HCl at 37°C, and three 5-min washes in PB at room temperature. Non-specific binding was blocked with 3% normal donkey serum (Jackson Labs) and 0.5% Triton X-100 in PB (“block”) for 1 h at room temperature, followed by 24–48 h exposure to sheep anti-BrdU (1:239, Capralogics) in block at 4°C. Sections were again rinsed in PB and then incubated overnight in biotinylated donkey anti-sheep IgG in PB (1:200, Vector Labs), followed by overnight incubation in streptavidin-conjugated Alexa 488 in PB (1:800, ThermoFisher Scientific) for visualization of BrdU. The next day, sections were washed with PB, blocked for one hour, and incubated overnight in mouse anti-Hu primary antibody at 4°C (1:200 in 3% block; Invitrogen). After three 5-min PB rinses at room temperature, tissue was exposed to donkey anti-mouse IgG conjugated to Cy-3 in PB (1:80; EMD Millipore) for 1 h for visualization of Hu. Finally, sections were washed, dehydrated with ethanols, delipidized with xylenes, and cover slipped with Krystalon (Millipore-Sigma).

### Microscopy

Data were collected without knowledge of bird identity, treatment, or brain hemisphere. Area measurements and cell counts for all regions were performed using a computer-yoked microscope and mapping software (Olympus BX51; Lucivid LED microprojection, Neurolucida, Microbrightfield, Inc.). The boundaries of regions of interest were traced in 6–12 sections per hemisphere per bird. Boundaries for HVC were established with dark-field optics based on neuropil density and contrast. Area X is found ventral and anterior to the densely reflective oval shaped lateral magnocellular nucleus of anterior nidopallium (lMAN) and is demarcated by a dense haze of terminals and small cells. Area X and lMAN are separated by lamina pallio-subpallialis (LPS) which is visible in darkfield as less reflective than the nuclei.

**Fig 2:**
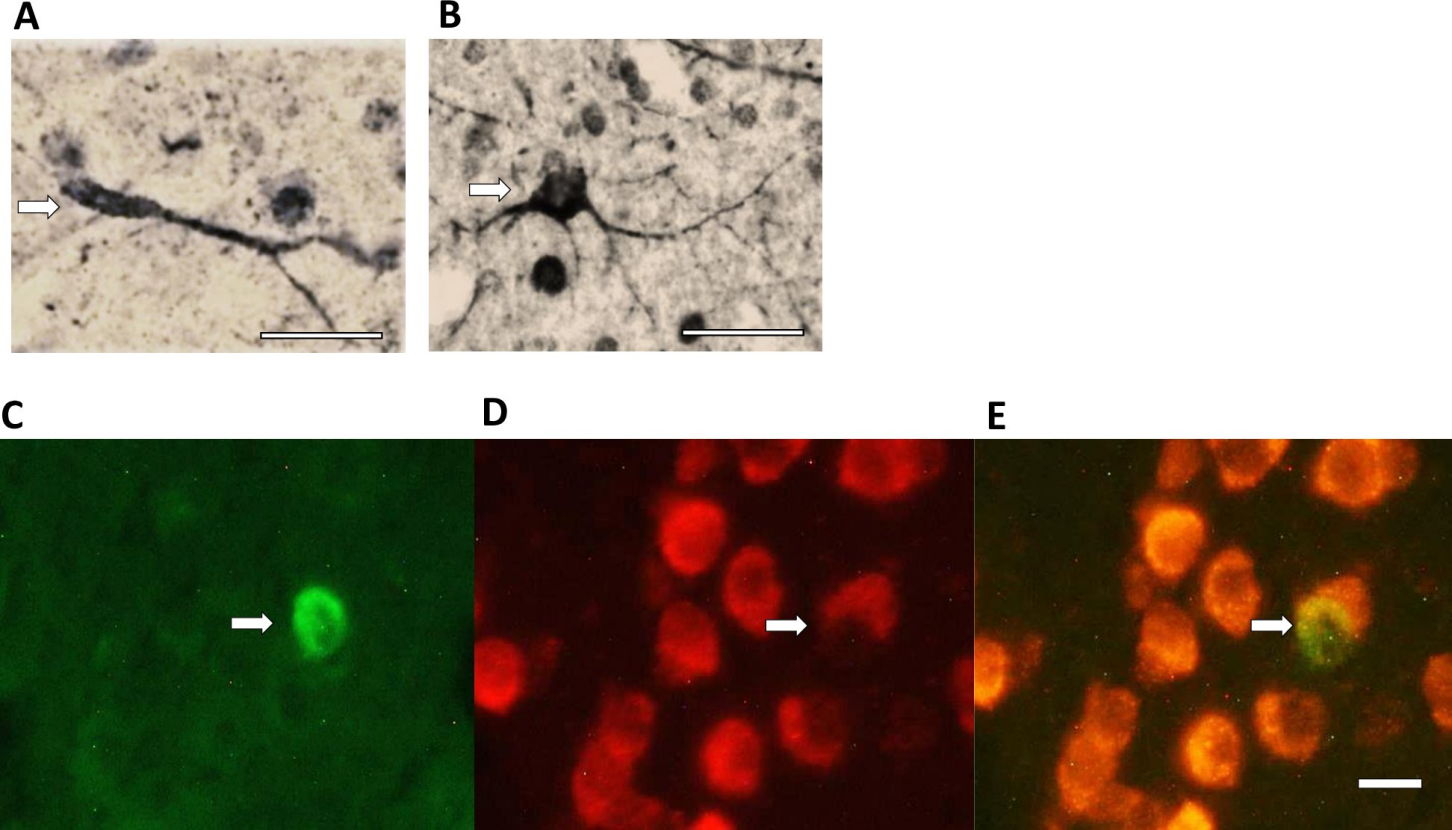
Photomicrographs of doublecortin-positive neurons in HVC. **A.** Fusiform, approximately 1–2 weeks old as indicated by its thin, elongated soma and unipolarity and **B.** Multipolar, approximately 2–3 weeks old as indicated by large, round soma and multiple processes (cell age estimates follow Balthazart, et al., 2008) [59]. **C.** Fluorescent markers used to identify 28–30 d old neurons in HVC, BrdU^+^ nucleus visualized with FITC filter. **D.** Hu^+^ neurons seen under rhodamine filter. **E.** BrdU^+^/Hu^+^ neuron showing colocalization of the two markers (rhodamine/FITC filter) in the same field of view. Scale bars A, B = 25 μm; C, D = 10 μm.

NCM was identified as in [57]. DCX^+^ cells were visualized with bright field light microscopy (Fig 2A and 2B). BrdU^+^/Hu^+^ cells were visualized using fluorescein isothiocyanate (FITC) and rhodamine filters and a dual FITC/rhodamine filter (Fig 2C–2E). New neurons per square millimeter was calculated by dividing the numbers of labeled cells by the total area sampled.

### Statistical analysis

Data are presented as means and SEMs unless otherwise specified. Analyses were performed using one-way ANOVAs, two-factor ANOVAs with repeated measures on hemisphere, and two-tailed *t*-tests for independent samples. Tukey’s HSD post-hoc tests were used following significant effects determined by ANOVA. Following significant mixed ANOVAs, we performed one-way ANOVAs between groups within individual hemispheres and then Holm’s procedure with adjusted p values for multiple tests. Pearson correlations were computed between cell counts across brain regions or hemispheres. The lateralization index (LI) indicates the relative number of new neurons between hemispheres, normalized for the bird’s mean number of new neurons per area sampled in both hemispheres (as in Tsoi et al., 2014) [24]. Higher positive values indicate more new neurons in the left hemisphere relative to the right, an outcome of arbitrarily subtracting right from left in the equation:

$$\frac{\text{Left}\;\text{neurons}/{\text{mm}}^{2}-\text{Right}\;\text{neurons}/{\text{mm}}^{\text{2}}}{\left(\text{Left}\;\text{neurons}+\text{Right}\;\text{neurons}\right)/\left(\text{Left}\;\text{area}\;\text{sampled}+\text{Right}\;\text{area}\;\text{sampled}\right)}$$

## Results

### Song analysis

**Fig 3:**
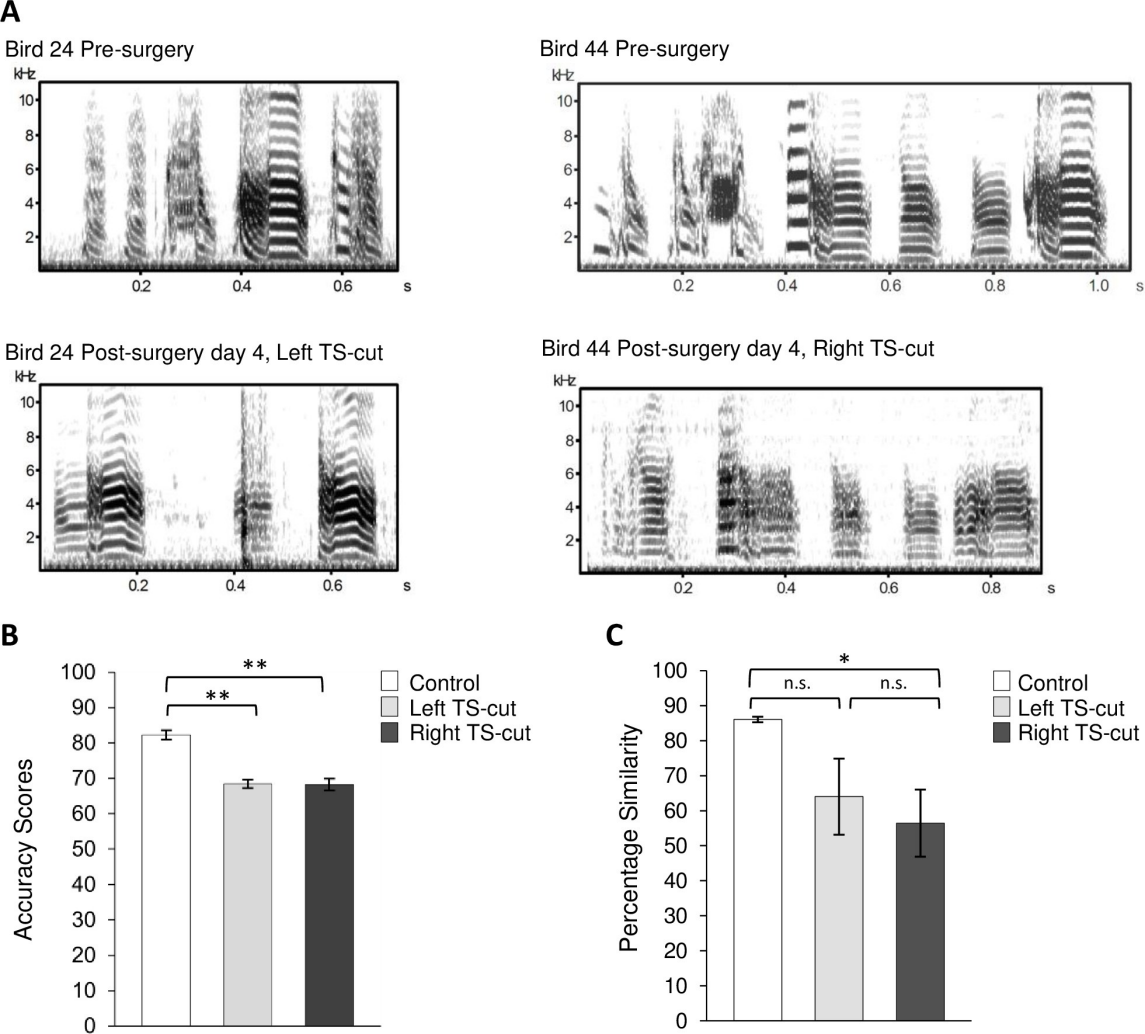
Effects of TS-cuts on song structure. **A.** Pre-operative spectrograms (top row) and post-operative spectrograms of left TS-cut (left) and right TS-cut (right) showing song changes after unilateral TS-cuts, recorded 4 days post-surgery. Changes in acoustic structure were highly varied across individuals. Spectrograms were generated with Sound Analysis Pro 2011 [56]. **B.** Birds that underwent left (n = 5) or right (n = 4) TS-cut had accuracy scores that were significantly lower than those of controls (n = 5), demonstrating that unilateral tracheosyringeal nerve cut impacted the quality of song production. **C.** There was a significant overall difference in the pre- to post- similarity scores among groups, and right TS-cut birds differed from controls. Control (n = 5), left TS-cut (n = 5), and right TS-cut birds (n = 4). Shown are means ± standard errors, * = p < 0.05, ** = p < 0.01, n.s. = p > 0.05.

Birds that underwent either a left or right TS cut (Fig 3A) had significantly lower accuracy scores, a measure of song change post-denervation, than did controls measured over the same time interval (One-way ANOVA, df = 2, F = 35.28, p < 0.0001, Fig 3B). Both left TS-cut and right TS-cut scores were lower than those of controls (Tukey’s post-Hoc test, p < 0.01) and there was no difference between the two denervated groups (Tukey’s post-Hoc test p > 0.05). There was also a significant difference in pre-to-postoperative similarity scores among groups (one way ANOVA, df = 2, F = 4.24, p = 0.043, Fig 3C). Similarity scores of the right TS-cut birds were significantly lower than those of control birds (Tukey’s post-Hoc test p < 0.05). Scores of the left TS-cut group did not differ significantly from either the right TS-cut or control birds. Likewise, changes in individual song features were not significantly different between right or left TS-cut groups (p > 0.05 for all: pitch, FM, entropy, goodness of pitch, AM; all data are available at: osf.io/486ez).

### TS-cuts did not impact new neurons in HVC

**Fig 4:**
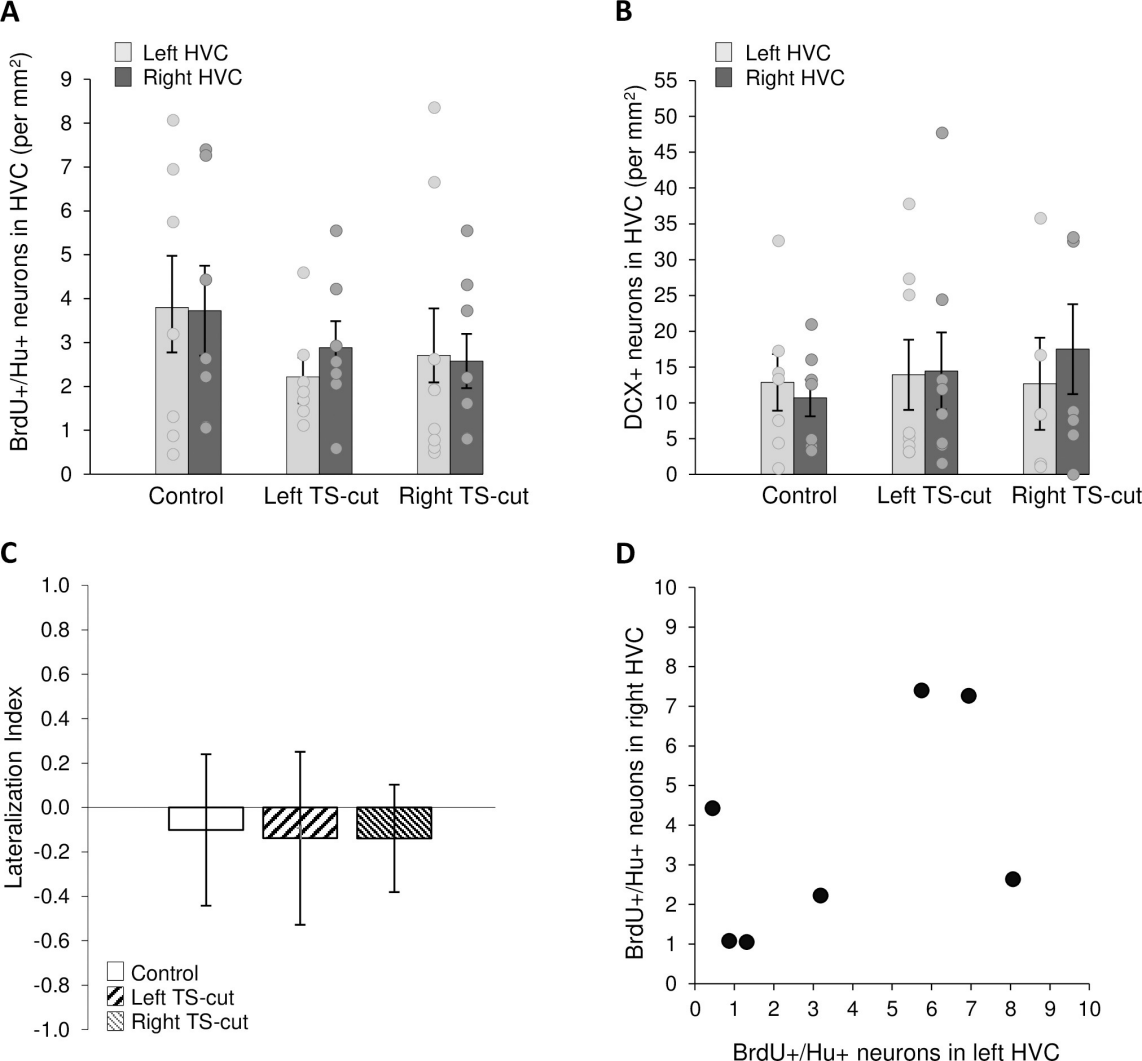
Numbers of new neurons in HVC as a function of hemisphere and experimental condition. **A.** Neither left (n = 7) nor right (n = 8) TS-cut affected the number of 28–30 d old BrdU+/Hu+ neurons in HVC compared to controls (n = 7). **B.** Neither left (n = 8) nor right (n = 5) TS-cut affected the number of ~1–3 week old DCX+ neurons in HVC compared with controls (n = 7). **C.** There were no differences in the Lateralization Index of BrdU+/Hu+ cells among treatment groups. **D.** There was no correlation between numbers of BrdU+/Hu+ cells between the left and right HVC in control birds.

There was no effect of treatment (df = 2, F = 0.75, p = 0.49), hemisphere (F = 0.04, p = 0.84), or interaction (F = 0.29, p = 0.75) on numbers of BrdU^+^/Hu^+^ cells in HVC that were 28–30 days old (two-factor ANOVA, repeated measures on hemisphere, Fig 4A). We also quantified DCX+ cells, a marker for immature neurons that is expressed for 2–3 weeks after mitosis in mammals [58] and was calculated to be expressed in newborn neurons for a similar time, approximately 20 days, in the canary [59]. The morphology of DCX+ neurons can also be used to estimate neuronal age in this time frame with fusiform, bipolar cells presumably with a migratory morphology being younger, and round, multipolar cells being older (Fig 2A and 2B; Balthazart et al., 2008 [59]). We did not find treatment or hemisphere differences when fusiform and multipolar cell counts were analyzed independently (p > 0.05 for all); therefore, we combined DCX+ cells. We found no effect of treatment (df = 2, F = 0.16, p = 0.85), hemisphere (df = 1, F = 0.05, p = 0.83), or treatment by hemisphere interaction (df = 2, F = 0.39, p = 0.68) on numbers of DCX+ cells in HVC (two-factor ANOVA, repeated measures on hemisphere, Fig 4B). We also found no difference in new neuron numbers (neither BrdU+/Hu+, nor DCX+) between hemispheres ipsilateral and contralateral to the TS nerve cut.

Unilateral TS-cuts had no effect on the lateralization index of BrdU+/Hu+ or DCX+ neurons in HVC (one-way ANOVA, df = 2, F < 0.01, p = 1.0; df = 2, F = 1.19, p = 0.33, respectively, Fig 4C). We also found no relationship between the numbers BrdU+/Hu+ neurons (Fig 4D) or DCX+ cells (not shown) in left HVC and right HVC of control animals (r^2^ = 0.53, t = 1.38, p = 0.23; r^2^ = 0.66, t = 1.1, p = 0.32, respectively).

Because TS-cuts did not affect numbers of new neurons in HVC, we were not surprised that there were no significant correlations between numbers of new neurons and postoperative changes in song acoustic structure. We examined both young neurons expressing DCX and ~1 month old neurons labeled with BrdU expressing Hu. There were no correlations between new neurons in HVC either ipsilateral or contralateral to the TS-cut and any of our acoustic difference scores measured between the pre- and post-operative songs (similarity, accuracy, pitch, FM, entropy, pitch goodness, or AM; left TS cut n = 5 and right TS cut n = 4, groups combined for regression). There were also no correlations between new neurons in left, right, or combined hemispheres of HVC and these acoustic difference scores.

### TS-cuts altered hemispheric lateralization of new neurons in NCM

**Fig 5:**
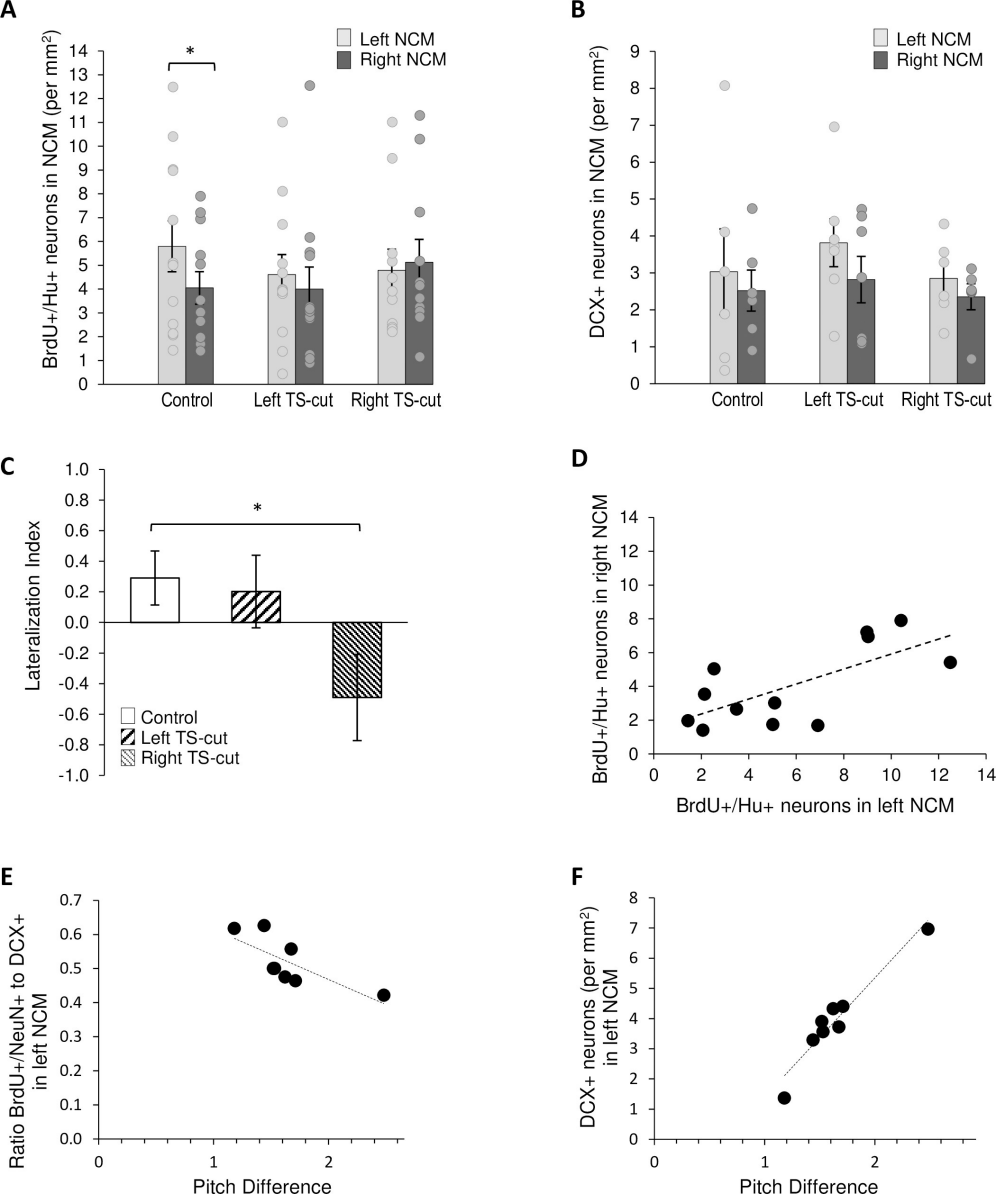
Numbers of new neurons in NCM as a function of hemisphere and experimental condition. **A.** Left (n = 12) and right (n = 11) TS-cuts had no effect on the overall number of 28–30 d old BrdU+/Hu+ neurons in NCM. Control birds (n = 12) had more BrdU+/Hu+ neurons in left NCM than the right and this asymmetry was not seen in the left or right TS-cut group. **B.** Neither left (n = 7) nor right (n = 6) TS-cut differed from controls (n = 7) in numbers of DCX+ neurons in NCM. **C.** The lateralization index of the right TS-cut group was significantly different from that of the controls; that of the left-TS cut group did not differ from controls or the right TS-cut group. **D.** There was a positive correlation between numbers of 28–30 d old neurons in left NCM and right NCM of control birds. **E.** We found a significant inverse correlation between the ratio of ~30 day old neurons (BrdU+/Hu+) to younger neurons (DCX+) and postoperative change in pitch (n = 4 left TS-cut and n = 4 right TS-cut with vocal recordings). The greater the change in pitch, the lower proportion of older neurons maintained out of the pool of younger neurons. Two data points are indistinguishable in the graph. **F.** There was a significant positive correlation between DCX+ neurons and the change in motif pitch (r^2^ = 0.925, F = 73.926, p = 0.0001; n = 4 left TS-cut, n = 4 right TS-cut birds with vocal recordings) This correlation was still significant without the highest value DCX+ data point (r^2^ = 0.878, F = 36.072, p = 0.002). * = p < 0.05.

There was not a main effect of treatment (F = 0.22, p = 0.80), hemisphere (F = 1.29, p = 0.27), nor treatment by hemisphere interaction (F = 0.83, p = 0.45) on numbers of BrdU+/Hu+ neurons in NCM (Fig 5A). As in HVC, we found no difference among treatments or hemispheres when DCX+ fusiform and multipolar cells were analyzed separately; therefore, we combined these categories. There was also no main effect of treatment (F = 0.48, p = 0.63), hemisphere (F = 2.28, p = 0.15), or interaction on DCX+ cells in NCM (F = 0.14, p = 0.87, two-factor ANOVA, repeated measures on hemisphere, Fig 5B).

Earlier work showed that in unmanipulated controls, there were more new neurons in the left NCM than the right, and that this asymmetry was lost after a unilateral tracheosyringeal nerve cut [43]. Here we also found more BrdU^+^/Hu^+^ neurons in the left than the right NCM in controls (paired sample t-test, t (11) = 2.26, p = 0.045; Fig 5A). As in Tsoi et al., (2014) [43] hemispheric asymmetry was not seen in either left TS-cut or right TS-cut groups (paired t-tests between hemispheres within groups, p = 0.67, p = 0.74, respectively). Consistent with this pattern, we found a significant difference in lateralization indices of BrdU+/Hu+ among the three treatment groups (df = 2, F = 3.92, p = 0.03). Interestingly, there was a greater effect of the right TS-cut than the left: the lateralization index of the right TS-cut group differed from controls (HSD, p <0.05) whereas the lateralization index of the left TS-cut group did not differ from that of controls or the right TS-cut group (Fig 5C).

Unlike in HVC, the numbers of BrdU+/Hu+ cells in left and right NCM of control birds were positively correlated (r^2^ = 0.69, t = 3.06, p = 0.01, Fig 5D) as in Tsoi et al. (2014) [43]. There were no correlations in numbers of new neurons between NCM hemispheres in either TS-cut group (r^2^ = 0.058, p = 0.45, left TS cut; r^2^ = 0.026, p = 0.63, right TS-cut. There were no correlations in numbers of DCX+ cells between NCM hemispheres in any group (p > 0.05 for all).

Because TS cuts decreased left-NCM dominance in ~30-day old neurons, we sought to identify acoustic parameters that correlated with the lateralization index. Side of TS cut did not affect the degree of change in acoustic feature (p > 0.05, comparing effects per feature between left- and right-TS groups). Therefore, we combined left and right TS cut groups for comparisons between degree of feature change and numbers of new neurons. The greater the post-operative change in pitch, the fewer ~30 day old neurons (BrdU/Hu+) relative to the numbers of younger neurons (DCX+) in left NCM (r^2^ = 0.568, F = 7.899, p = 0.031, Fig 5E; right NCM > 0.05, not shown). This ratio may be more intuitively thought of as the numbers of young (DCX+) cells that survive to day ~30 (BrdU+/Hu+).

We then looked at correlations between changes in acoustic features and DCX+ and BrdU+/Hu+ neurons individually. Change in pitch was positively correlated with numbers of young DCX+ neurons in the left NCM (r^2^ = 0.925, F = 73.926, p = 0.0001, Fig 5F; right NCM > 0.05, not shown). Change in pitch did not correlate with numbers of BrdU+/Hu+ cells in either hemisphere (p > 0.05). Together, this suggests that the postoperative change in song pitch increased recruitment of new neurons in the left hemisphere, but not their survival. No one particular acoustic feature that we measured accounted for the postoperative loss of left hemisphere dominance in new neurons of the BrdU+/Hu+ cohort. No other measure of change in song was correlated with numbers of new neurons in left or right NCM.

### TS-cuts decreased new neurons bilaterally in Area X

**Fig 6:**
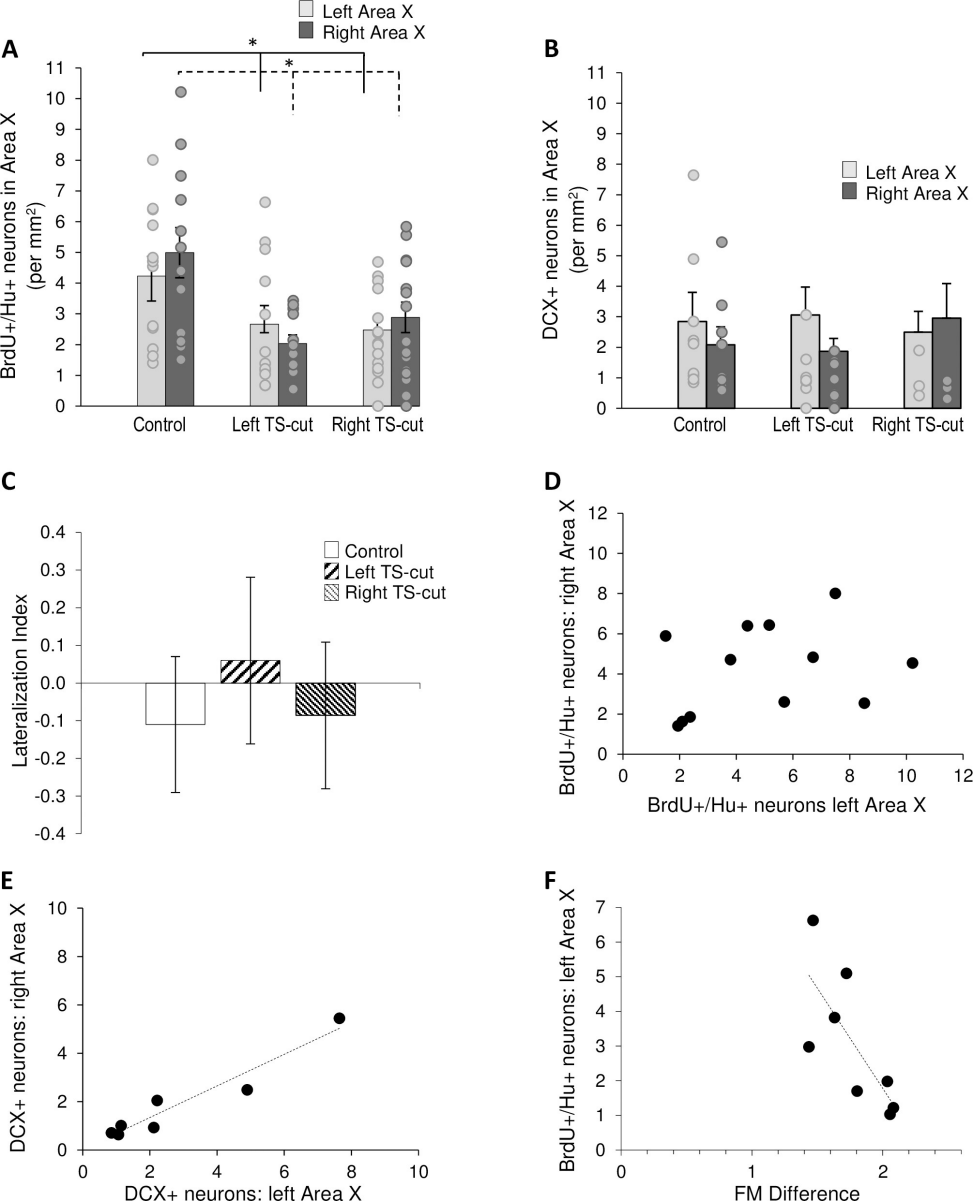
Numbers of new neurons in Area X as a function of hemisphere and experimental condition. **A.** Left (n = 12) and right (n = 14) TS-cuts significantly decreased the numbers of 28–30 day old neurons compared to controls (n = 12). Control birds had significantly more new neurons in the left hemisphere compared to the left hemispheres of the left and right TS-cut birds (solid lines). Similarly, control birds had significantly more new neurons in the right hemisphere than did left TS-cut and right TS-cut birds (dashed lines). **B.** Neither left (n = 9) nor right (n = 8) TS-cut groups differed from controls (n = 7) in numbers of DCX+ neurons in Area X. **C.** Lateralization indices for BrdU+/Hu+ neurons in Area X. LIs did not differ between controls, left TS-cut, and right TS-cut conditions. **D.** There was no relationship between the numbers of BrdU+/Hu+ neurons in left versus right Area X in controls. **E.** We found a significant correlation between left and right Area X in numbers of DCX+ neurons in controls. **F.** The degree of change in song frequency modulation post-TS cut was significantly inversely correlated with numbers of BrdU/Hu+ neurons in left Area X (n = 4 left TS-cut and n = 4 right TS-cut birds with vocal recordings). * = p < 0.05.

We found a significant difference in the number of BrdU+/Hu+ neurons in Area X among the three treatment groups (two factor repeated measures ANOVA, F = 7.47, p = 0.002, Fig 6A). There was no difference between hemispheres (F = 0.25, p = 0.62) and no interaction between treatment and hemisphere (F = 1.16, p = 0.33). Within the left hemispheres (one way ANOVA, df = 2, F = 3.31, p = 0.048), there were fewer BrdU+/Hu+ cells in both the left TS-cut and right TS-cut groups compared to controls (t = 2.05, p = 0.048; t = 2.39, p = 0.045, respectively). The same pattern was found within the right hemispheres of Area X (one way ANOVA, df = 2, F = 6.84, p = 0.003). Compared to the right Area X of controls, there were fewer BrdU+/Hu+ cells in the right Area X of both the left TS-cut and right TS-cut groups (t = 3.58, p = 0.002; t = 2.65, p = 0.012, respectively).

As in the other regions, we combined all DCX+ cells after finding no difference between fusiform and round DCX+ cell types assessed separately across treatments. There was no main effect of treatment (2 factor mixed ANOVA, F = 0.07, p = 0.93), hemisphere (F = 1.48, p = 0.24), or interaction (F = 1.22, p = 0.32; Fig 6B) on the numbers of DCX+ neurons in Area X.

There was no significant difference among groups in the Lateralization Index of BrdU+/Hu+ or DCX+ cells (one-way ANOVA, F = 0.2, p = 0.82; F = 0.20, p = 0.82, respectively, Fig 6C). There were no correlations between the numbers of BrdU+/Hu+ neurons in the left and right Area X of control animals (r^2^ = 0.30, p = 0.35; Fig 6D). However, DCX+ cells were positively correlated between hemispheres in control birds (r^2^ = 0.92, p = 0.016, Fig 6E) and in birds with a left side TS-cut (r^2^ = 0.85, p = 0.03), but not in birds with a right side TS-cut (r^2^ = 0.67, p = 0.27).

We found an inverse correlation between the degree of change in FM and the numbers of 28–30 day old neurons in Area X in the left hemisphere (r^2^ = 0.565, F = 7.808, p = 0.031, Fig 6F) and both hemispheres combined (r^2^ = 0.581, F = 8.329, p = 0.028, n = 4 left TS-cut and 4 right TS-cut). Despite the decrease in new neurons of TS-cut birds in each hemisphere, no other measure of change in song was correlated with neuron numbers in left or right Area X.

### No correlations in numbers of new neurons between regions within hemispheres

We were also interested in whether new neuron numbers may be linked between functionally associated regions in control birds. However, we found no correlations in numbers of BrdU+/Hu+ cells between NCM and HVC or between HVC and Area X in either the left (r^2^ = 0.69, p = 0.34; r^2^ = 0.89, p = 0.063, respectively) or right (r^2^ = 0.69, p = 0.35; df = 5, r^2^ = 0.49, p = 0.61, respectively) hemispheres. There were no correlations between DCX+ cells within either hemisphere between NCM and HVC (left: r^2^ = 0.48, p = 0.340; right: r^2^ = 0.47, p = 0.35) or between HVC and Area X (left: r^2^ = 0.79, p = .063; right: r^2^ = 0.23, p = 0.612).

### No correlations in numbers of new neurons and bird age

There were no correlations between bird age at the time of BrdU injections and numbers of new neurons in either the left, right, or combined HVC (F = 0.009, r^2^ = 0.001, p = 0.926; F = 0.002, r^2^ < 0.001, p = 0.965; F = 0.191, r^2^ = 0.027, p = 0.675; respectively). Similarly, there were no correlations between bird age at the time of BrdU injections and numbers of new neurons in either the left, right, or combined NCM or Area X, (p >0.6 for all).

## Discussion

This is a broad-strokes study, serving as a first pass in understanding whether the quality of song structure affects new neuron survival and whether regulation of new neuron survival is independent across brain regions and hemispheres. We found that irreversible disruption to song structure by unilateral denervation of the syrinx in adult male zebra finches impacted the numbers of 28–30 day old neurons in a region and hemisphere-specific manner. Altered song feedback had no effect on numbers of one month old neurons in either hemisphere HVC, resulted in a loss of left-sided dominance of new neurons in NCM consistent with Tsoi et al. (2014) [43], and decreased neuron survival in both hemispheres of Area X. These effects were not present in younger neurons expressing DCX, perhaps due to a lack of functional connectivity in this cohort at the time of treatment. In earlier work, we posited that either song quality or song recovery influenced new neuron survival in HVC [42]. Here we suggest that song quality in itself does not influence new neuron recruitment (of DCX+ cells) or new neuron survival (of 28–30 day old BrdU+/Hu+ cells) in HVC. These negative results therefore lend support to the idea that either song recovery promotes new neuron survival in HVC or vice versa [42]. Larson et al. (2013) [14] showed that RA activity promotes new neuron survival in HVC in adult male Gambel’s white crowned sparrows, using unilateral muscimol infusion into RA. Interestingly, the treatment also altered song structure, allowing an assessment of sensory factors that may impact new neuron survival. Consistent with our results, they found no correlation between the degree of song degradation and the number of new neurons in HVC either ipsi- or contralateral to the side of infusion. Together, these findings increase support for the idea that new neuron addition to HVC may be insensitive to song-related sensory feedback.

### What does the TS nerve section do?

The aspects of the TS nerve cut that affected new neuron survival in Area X and lateralization in NCM are not known. In addition to changing the acoustic structure of vocalizations, TS-nerve sectioning also would alter putative somatosensory feedback from syringeal muscles [60,61] and potentially somatosensory feedback from the air sac receptors if bronchial resistance was changed by syringeal denervation [62]. It is not known whether somatosensory information from the syrinx feeds back into NCM or the song system thereby providing an opportunity to impact the brain regions examined. But it has been proposed that this may occur via syringeal afferents to the caudolateral part of the interpolaris subdivision sensory trigeminal nuclei (nTTDi) via nucleus uvaeformis [61,63]. TS nerve cuts may also have altered the rate of song production, as did botox injections within the first week after treatment [42]. The quantity of song output corresponds to numbers of new neurons in canary HVC [20,21,24,64] but this has not yet been found in zebra finches [42,65]. To our knowledge, there is no information about whether singing rate impacts numbers of new neurons in song regions other than HVC in any species.

More associations between measures of acoustic change and numbers of new neurons in NCM and Area X would have better supported the idea that mismatched acoustic feedback influenced the survival of new neurons in these regions. Regardless, it is intriguing that the postoperative change in pitch was inversely correlated with numbers of new neurons in left NCM and the postoperative change in FM was inversely correlated with numbers of new neurons in Area X. In sum, we cannot rule out the possibility that the TS nerve cuts brought about the change in new neurons in Area X by decreasing singing rate, or through an interaction between singing rate and altered acoustic structure.

### New neurons in NCM are sensitive to TS nerve cuts

We speculate that the change in new neuron lateralization in the experimental birds may be due to a mismatch between expected and received acoustic feedback. Neurons in NCM respond to playback of the bird’s own song as well as the song of the bird’s tutor and other conspecifics [66,67]. Moreover, NCM is active while a bird is singing, suggesting it is not gated during singing and may process auditory feedback from song in real time [36]. In particular, motor recovery from experimental distortion of song pitch requires an intact NCM, identifying a role for NCM in storage and/or recall of at least this feature of a bird’s own song [68]. As such, mismatched pitch may lead to fewer new neurons being maintained during early culling of the new neurons recently arriving in NCM.

NCM receives a robust influx of new neurons throughout adulthood, although very little is known about the function of these new neurons. We do know that new neurons are upregulated in birds housed in groups [30,31,69,70] and are diminished in birds that have been deafened [57]. Together, this suggests that activity in NCM promotes the maintenance of new neurons; however, does not shed light on their function. Generally, the continual addition of new neurons may preserve and/or improve the resolution of stored song memories.

A comparison between expected and received feedback requires a memory of the bird’s own song. Because the effect of the TS cut on new neurons was lateralized, perhaps NCM is functionally lateralized in performing this comparison. We found that numbers of new neurons were greater in the left than right hemisphere in control birds, and that this asymmetry was lost in TS cut bird. Both findings are consistent with Tsoi et al., (2014) [43]. The loss of asymmetry was due more to a decrease in numbers of new neurons in the left NCM than an increase in new neurons in the right NCM, although the degree of difference was not statistically significant. One of several explanations for this finding is that the new neurons in the left hemisphere may be more sensitive to feedback mismatch, and may be so if a memory of expected feedback is lateralized to the left, rather than right, NCM. Although a comparison need not take place in the same hemisphere as the new neuron effect, it is a parsimonious explanation.

Evidence for a lateralized response in NCM to the bird’s own song is mixed. It is suggestive that estradiol synthesis in the left NCM but not the right NCM is necessary for a bird’s preference for his own song [71]. In a behavioral study, adult male zebra finches showed deficits in discriminating their own song from that of a cage-mate when the left side ovoidalis, part of the ascending auditory pathway, was lesioned and not when the right side was lesioned [72]. However, selectivity for playback of the bird’s own song was found to be lateralized toward the right side in the midbrain dorsal lateral mesencephalic nucleus (MLd), the analog of the mammalian inferior colliculus [73]. Like ovoidalis, MLd is part of the unilateral ascending auditory pathway that eventually projects to NCM. Moreover, both left and right NCM responded to playbacks of the bird’s song, although not selectively over conspecific song, and there was no evidence of hemispheric asymmetry [74].

In a different model, hemispheric asymmetry may be based on specializations for sound acoustic features rather than categories such as own song versus those of others [75]. Playback of conspecific songs with filtered spectral structure increased blood-oxygen level dependent (BOLD) fMRI responses in the left NCM and slightly decreased BOLD responses in the right NCM compared to nonmanipulated songs [75]. Because spectral filtering removed frequency information from the song leaving primarily temporal content, the authors interpreted this finding as indicating that the left NCM is specialized for processing temporal information. They suggested that this asymmetry is normally masked by the spectral content of whole songs [75]. On the other hand, we suggest that perhaps the increased response in the left NCM reflects a left-dominant sensitivity to aberrant information, resonating with our findings. The left hemisphere of NCM has been shown to be sensitive to novel auditory experience more broadly [76,77] consistent with the idea that NCM (in these studies, bilaterally) is sensitive to expectation [78,79]. Thus perhaps it is the novelty of the distorted song, and not feedback about the bird’s own song *per se*, that underlies the observed decrease in new neurons in the left NCM.

It is a consistent finding across studies that the magnitude of asymmetry in different metrics of NCM corresponds to performance in learning [80]. For instance, the degree of left side dominance in new neurons [43], and left side dominance in activity during sleep [77] predict accuracy of song learning of the bird’s own song. Left side dominance in NCM activity in response to song playback is also correlated with rate of learning in a conspecific song discrimination task [81]. The results of the current study and that of Tsoi et al., (2014) [43] add that lateralization of new neurons can be influenced by the bird’s experience.

### Is the decrease in new neurons in Area X mediated by dopamine?

Dopamine is a likely candidate for mediating new neuron survival in Area X in response to altered song acoustic structure. Area X plays a role in juvenile song learning [34,35,82], and in modifying the adult song motor pattern in response to social context, deafening, and perturbations in feedback [37,83–89]. New neurons are continually added to Area X, increasing cell packing density with increasing bird age [90], as occurs in HVC [28]. Approximately 80% of new neurons become medium spiny neurons (MSN), express D1 and D2 receptors, and fire during singing [90], although the contribution of new neurons in particular is not known.

Dopamine promotes new neuron survival in the mammalian hippocampus and in the subventricular zone via D1 and D2 receptor activation [91,92] and is a known modulator of activity in Area X. The substantia nigra pars compacta (SNc) and the ventral tegmental area (VTA) send direct dopaminergic projections to Area X [93–95]. Through evaluation of predicted and actual auditory feedback, dopaminergic signals modulate the output of the anterior forebrain pathway [96–102].

HVC neurons that project to Area X encode own-song acoustic information and respond to delayed auditory feedback with aberrant firing, suggesting dynamic input of own-song acoustic information into Area X [103]. This is consistent with earlier work showing that dopaminergic neurons in Area X are active in response to playback of the bird’s own song [104] and thus are poised to evaluate own song feedback and respond with dopamine release. More precisely, Gadagkar et al., (2016) recorded dopaminergic VTA neurons that project to Area X while birds were singing normally, and also while singing as experimenters played back distorted song syllables timed to be interpreted as own-song errors [105]. They found that VTA firing was suppressed after distorted syllables, and concluded that error signals can be encoded in the dopamine response. This is important for many reasons, but relevant to our findings it shows that DA modulation is a read out of the bird’s perceived vocal success based on an internally encoded, or expected, goal. Similarly, decreasing VTA activity in Area X via 6-OHDA-induced retrograde lesions of dopaminergic cells in the VTA resulted in loss of the vocal learning that is normally driven by disruptive auditory feedback in a negative reinforcement task [106,107]. The association between DA and encoding of altered auditory feedback was further tested via optogenetic inhibition and excitation of VTA axon terminals during singing, modulating DA in Area X. DA modulation predictably guided changes in targeted song syllable production consistent with positive and negative reinforcement of performance [101]. Moreover, externally reinforced vocal pitch learning relies on the same VTA-Area X pathway as internally guided vocal copying in juvenile zebra finches [108]. Thus it is suggestive that we found an inverse correlation between the degree of song change specifically in frequency modulation and numbers of new neurons in Area X. Area X is also known to modulate the production of song spectral features but not temporal features, and to do so via activation of D1 receptors [85,108,109]. However, we cannot account for the effect being limited to the left, and not in both hemispheres, as there are no reports of asymmetries in Area X function.

Interestingly, the influence of the VTA on Area X is reciprocal. Area X makes an indirect connection to SNc/VTA via a projection to ventral pallium (VP) [94]. Through this pathway, Area X output drives changes in the firing rate of dopaminergic neurons in the SNc and VTA. VP neurons are spontaneously active and, notably, are preferentially inhibited by BOS, suggesting that Area X disinhibits dopaminergic neurons by inhibiting VP [104]. This relationship suggests that Area X plays a role in feedback evaluation of song with respect to an internal model of the bird’s own song, and transmits this information to dopaminergic neurons. Perhaps this pathway results in decreased dopamine signaling in the TS-cut birds with mismatched feedback, and fewer new neurons, in Area X.

### There were no correlations in numbers of new neurons between regions

Few studies in any species have made inter-regional comparisons of adult neurogenesis with an aim toward identifying region-specific regulatory mechanisms. Notably, prenatal stress has been shown to decrease neurogenesis in the mouse hippocampus in adulthood, with no effect on neurogenesis in the olfactory bulb [8]. In the aging pigeon, the extent of new neuron decline is greater in the olfactory bulb than in the hippocampus, again differentiating regulatory mechanisms between neurogenic regions [110]. Rates of decline in new neurons during aging also differ between HVC and Area X [53]. Consistent with these findings, our results also suggest that regulatory mechanisms may have independent components across brain regions, even those linked functionally and anatomically.

### There were no correlations in numbers of new neurons and bird age

Although our birds spanned the ages of 4–11 months, the majority of animals clustered at 4, 5, and 7 months of age at the time of the brdu injections, equally represented in the 3 groups, and this likely contributed to our finding that age was not a factor in our cell counts. Earlier work found no effect of age on new neuron numbers in HVC in zebra finches between the ages of 4–8 months (Wang et al., 2002) [14]. A decrease in new neurons was evident only when comparing birds younger than a year with those 12 mos or older, and an age-related correlation in new neurons required comparing birds across ages that spanned the period of 8–14 mos (Wang et al., 2002) [14] or between groups aged 3–5 months old and 14–21 month (Pytte et al., 2007) [53]. Therefore, the upper age range of our birds was also likely not high enough for age to be a correlate. Our finding of no effect of age in Area X and NCM is also consistent with the few reports in the literature. There was no difference in numbers of new neurons added to Area X in a grouped comparison between zebra finches 3–5 months old and those 14–21 months old (Pytte et al., 2007) [53]. Likewise, it was reported in starlings that there was no difference in new neurons in NCM in birds grouped as yearlings, 2^nd^ year, and 3 years and older [111]. Ring doves, on the other hand, demonstrated an age-related decline in new neurons added to the caudal nidopallium at about 3 months and 1 year of age [112]. Given the well-documented age-related decline in hippocampal neurogenesis in mammals [113], this is likely a common principal; however, not one captured by our study.

### Taken together…

Song learning by juveniles requires incremental improvement toward a target motor behavior and requires both Area X and HVC, in concert with neuronal tuning to the tutor and the bird’s own song in NCM. Moreover, song learning occurs at a time when new neuron incorporation is high. In one model of song learning, akin to procedural motor learning more broadly, Area X and the anterior forebrain pathway is thought to evaluate feedback and influence subsequent motor commands produced by HVC, and the output of feedback evaluation is thought to influence subsequent motor commands. Our findings suggest that the accumulation of new neurons in Area X may correspond directly to the quality of song feedback in matching to the target own-song template. As in bilateral Area X, new neurons in left NCM may likewise be culled when song feedback no longer matches expected feedback of the bird’s own song. On the other hand, new neurons in HVC seem to not be culled or maintained based on the output of a match between expected and received feedback. New neurons in HVC may instead 1) be influenced via feedback with respect to progress toward a goal of an ideal song or 2) not be affected by song feedback at all, and instead new neurons may drive progress toward a goal song in a feedforward direction only (both ideas consistent with Pytte et al., 2011) [42]. Here we eliminate the hypothesis that the quality of song per se influences HVC new neuron survival. In sum, perhaps the continual addition of new neurons in the song system, and perhaps in NCM also, increases the resolution of these neuronal substrates which in turn achieves increased song stereotypy both by better precision of the evaluation of song feedback (NCM and Area X) and by fine tuning motor output (HVC).

## Conclusions

This study demonstrates that altered feedback affects new neuron recruitment and survival in brain regions that subserve and monitor those behaviors. We found that altering song production via unilateral tracheosyringeal denervation resulted in decreased 28–30 day old neurons in Area X, loss of lateralization of neurons in NCM, and had no effect in HVC. This indicates that the effects of syringeal denervation on neurogenesis vary depending both on the region and hemisphere that receives new neurons.
